# Editorial: Linking experimental and computational connectomics

**DOI:** 10.1162/netn_e_00108

**Published:** 2019-09-01

**Authors:** Alexander Peyser, Sandra Diaz Pier, Wouter Klijn, Abigail Morrison, Jochen Triesch

**Affiliations:** SimLab Neuroscience, Jülich Supercomputing Centre (JSC), Institute for Advanced Simulation, JARA, Forschungszentrum Jülich GmbH, Jülich, Germany; SimLab Neuroscience, Jülich Supercomputing Centre (JSC), Institute for Advanced Simulation, JARA, Forschungszentrum Jülich GmbH, Jülich, Germany; SimLab Neuroscience, Jülich Supercomputing Centre (JSC), Institute for Advanced Simulation, JARA, Forschungszentrum Jülich GmbH, Jülich, Germany; SimLab Neuroscience, Jülich Supercomputing Centre (JSC), Institute for Advanced Simulation, JARA, Forschungszentrum Jülich GmbH, Jülich, Germany; Institute of Neuroscience and Medicine, Institute for Advanced Simulation, JARA Institute Brain Structure-Function Relationships, Forschungszentrum Jülich GmbH, Jülich, Germany; Institute of Cognitive Neuroscience, Faculty of Psychology, Ruhr-University Bochum, Bochum, Germany; Frankfurt Institute for Advanced Studies, Johann Wolfgang Goethe University, Frankfurt am Main, Germany

**Keywords:** connectomics, brain structure to function, interdisciplinary neuroscience, multiscale, high performance computing

## Abstract

Large-scale in silico experimentation depends on the generation of connectomes beyond available anatomical structure. We suggest that linking research across the fields of experimental connectomics, theoretical neuroscience, and high-performance computing can enable a new generation of models bridging the gap between biophysical detail and global function. This Focus Feature on ”Linking Experimental and Computational Connectomics” aims to bring together some examples from these domains as a step toward the development of more comprehensive generative models of multiscale connectomes.

An important direction for theoretical and experimental neuroscience is the development of in silico experimentation through the use of simulation and analysis of large-scale and biologically realistic neuronal networks (Einevoll et al., 2019) in order to predict the functional and clinical consequences of brain structure. The connectome, the ensemble of anatomical connections between neurons (Sporns et al., 2005), is a fundamental player in the production of brain function. Large-scale connectome data defining circuits internally and linking them to other circuits and brain regions is essential for modeling biologically realistic networks.

Current computational infrastructure allows us to simulate models with increasingly larger and more detailed connectomes, as well as to investigate connectivity data extracted from experiments with increasingly higher precision and complexity, as seen in the Human Brain Project (Amunts et al., 2016) and the Brain Initiative (Mott et al., 2018). However, anatomical structures in connectomic datasets are incomplete and contain large uncertainties (Bakker et al., 2012), while functional data cannot fully specify unique connectomes—the structure/function relationship is not uniquely invertible. How can we go from incomplete structural data to predicted functional behavior to enable in silico experimentation?

One way to address this challenge is the generation of neural connectivity, for example, through either simulated individual development (Nowke et al., 2018) or simulated evolutionary processes (Avena-Koenigsberger et al., 2014; Bellec et al., 2018). The scientific infrastructure to enable the construction of connectomes at either level may involve generative connectivity rules, homeostatic and plasticity rules transforming functional networks, detailed morphological data perturbed to fit behavioral outputs, and the improvement of simulator technology to couple all these elements of connectome construction and to enable direct interaction by scientists using visualizing technology. To catalyze the combination of such interdisciplinary development, a satellite workshop at the 2017 Bernstein Conference in Göttingen, Germany, on “Connectivity generation, exploration and visualization for large-scale neural networks” was organized, spanning generative rules, simulator integration, visualization, mapping of experimental data to connectome generation and dynamic processes at multiple timescales.

The presentations covered both technical and scientific questions at multiple organizational levels: How should an abstract representation of connectivity be derived from experimental data? Which connectivity elements are essential for characterizing different levels of organization? What is the interplay between changes in connectivity and slow processes like neuromodulation, neurodegeneration, and neurodevelopment? What are the essential features of connectivity that impact function, at which scales, and to which extent? A lack of definite answers to these questions has limited our ability to exploit the extremely complex data acquired through experiments in models of neural networks for simulation and analysis.

Over two days, 11 presentations by workers in the fields of network neuroscience, computer science, high-performance computing and experimental neuroscience helped to elucidate some answers to these challenges. Scientists attending from throughout Europe ranged from master’s students entering the field to renowned senior scientists. In this Focus Feature on “Linking Experimental and Computational Connectomics,” we present a selection of work derived from these discussions from both theoretical and experimental points of view.

Hilgetag et al. (2019) give us a panoramic view on our current knowledge of cortical connectivity in the primate brain. With their analysis of fundamental structural parameters in different cortical areas, they demonstrate the existence of an architecture that can explain and predict the structural organization of the brain across scales.

Whether during development, learning, adaptation, input integration, disease, or recovery after lesions, the connectivity in the brain is always changing. Lu et al. (2019) explore the impact of external stimulation on the brain’s structure for therapeutic purposes and provide initial insight on the inner mechanisms regulating long-term functional recovery by connectivity rewiring.

Hailing from the experimental methods side of the field, Rojas et al. (2019) introduce a framework based on wavelet transform methods and apply it to the analysis of complex multiscale dynamic events in in vivo long-term loose patch clamp recordings of the cockroach circadian clock circuits. This novel toolset will enable the next step in the detection of correlations in multiscale neuronal behaviors and thus advance functional connectomics.

Interdisciplinary venues such as this workshop, training events, and journals in the spirit of *Network Neuroscience* promote progress across the divide between experimental, theoretical, and computational connectomic neuroscience. Collaboration across these disciplinary domains is critical toward developing a multiscale understanding of neuronal networks capable of bridging the gap between local biochemical details on the timescale of milliseconds and global plasticity and behavior occurring over hours and even the entire life span. The rise of generative connectomics—unifying experimental, theoretical, and computational approaches—is an important step toward grappling with such challenges of scale in the face of necessary limits in experimental constraints, requiring collaboration across neuroscience and beyond.
